# Development and Implementation of an Emergency Medicine Podcast for Medical Students: EMIGcast

**DOI:** 10.5811/westjem.2015.9.27293

**Published:** 2015-10-22

**Authors:** Andrew Lichtenheld, Mari Nomura, Nicholas Chapin, Trenton Burgess, Joshua Kornegay

**Affiliations:** Oregon Health & Science University, Department of Emergency Medicine, Portland, Oregon

## INTRODUCTION

Podcasts, episodic digital audio recordings downloaded through web syndication or streamed online, have been shown to be an effective instructional method in undergraduate health professions education, and are increasingly used for self-directed learning.^1^–^6^ Emergency medicine (EM) has embraced podcasting: over 80% of EM residents report listening to podcasts and a substantial number identify podcasts as the most valuable use of their educational time.^4^ Despite proven efficacy in undergraduate medical education and remarkable popularity with EM residents and attendings, there remain few EM podcasts targeted to medical students.^5^ Given that podcast effectiveness correlates with how well content matches the listener needs, a podcast specific to EM-bound medical students may optimally engage this target audience. 6

## OBJECTIVES

Our educational goals involved both content and process: 1) to produce a podcast delivering core EM content targeting specific needs of medical students interested in EM; and 2) to provide EM-bound medical students an opportunity to develop the ability to use podcasts as a self-directed educational modality. We measured time, costs, and resources as feasibility data and tracked podcast uptake as an initial measure of learner acceptability.

## CURRICULAR DESIGN

We developed this educational advance based upon established instruction design methods:

### Problem Identification, General and Targeted Needs Assessment

Our student EM interest group (EMIG) identified a gap in podcasts meeting learning needs of EM-bound medical students through peer discussions. A general needs assessment included a literature search and review of existing podcasts for medical students and with EM content, and informed a targeted needs assessment comprised of discussions with students and physicians regarding students’ needs and interests.

### Goals and Objectives

We designed the curriculum to prepare medical students to:

1. Demonstrate the ability to access podcasts and incorporate them into strategies for self-directed learning.
2. Develop familiarity with important themes and terminology in clinical EM practice.
3. Analyze and summarize discussions of core EM content in order to apply it to clinical practice.
4. Specific learner objectives were also developed for individual podcast.

### Educational Strategies

We chose the podcast modality for its effectiveness, popularity and accessibility.^4^ Under EM faculty guidance, a group of four medical students identified EM topics of importance to their peers. Content categories included: clinical conditions encountered in EM; logistics of applying to residencies, training, and working in a career in EM; and ethics encountered in EM. After defining a content outline, targeted learner objectives were developed. Medical students then identified content experts consisting of EM residents, nurses, and faculty and invited them to participate in student-led interviews. For each podcast an interview protocol was created with an anticipated discussion flow structured to elicit content matching learner objectives.

### Implementation, Resources and Logistics

Portable audio recording equipment and an online blog and podcasting platform were purchased with funds obtained from an educational grant. Startup equipment and costs totaled $400. We branded our podcast “EMIGcast,” reflecting the involvement of EMIG. Five episodes were developed, recorded, edited for clarity and brevity (average final length 33 minutes), and web-syndicated through the EMIGcast website and iTunes over a five-month period.^7^ Software with the podcasting platform was used to capture feasibility data including downloads of the audio content.

## IMPACT/ EFFECTIVENESS

We present the development and implementation of a podcast specifically designed to achieve content and process goals targeting EM-bound medical students. We found the podcast feasible to implement and acceptable to our learners. Data collected from the first 20 weeks of the “EMIGcast” podcast demonstrates an average of 148.5 downloads per month (698 total downloads) with a consistent increase in monthly downloads (Figure). While the absolute number of downloads is modest, the upward trend suggests growing acceptability and the number of downloads represents a scalability exceeding what we have previously achieved with EMIG lectures and panel discussions. We have also seen that episodes continue to be downloaded months after release, implying that the asynchronous and longitudinal availability of the podcast may be valuable to students over time. The student-initiated format may help with learner buy-in and the affiliation with EMIG provides name recognition and an inherent benefit for sustainability. We plan to identify new student leaders in incoming EMIG members each year and use consistent longitudinal faculty leadership to maintain institutional history and adherence to the overarching goals of the intervention. Future planned evaluations of the podcast include survey of listeners to better measure success in achieving learning objectives and to better characterize the educational impact of this educational intervention.

## Figures and Tables

**Figure:**
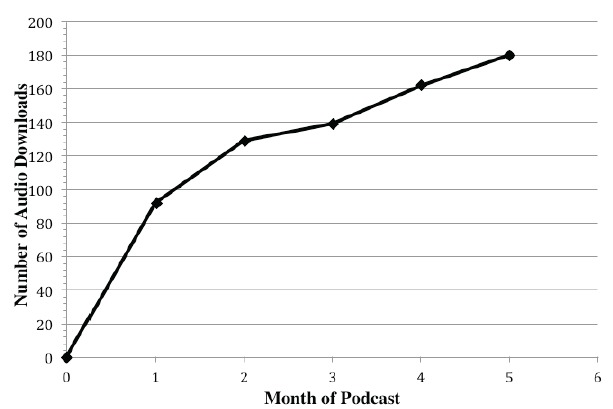
Total downloads by month of “EMIGcast” syndication
